# 3-(3-Bromo­phen­yl)-*N*-phenyl­oxirane-2-carboxamide

**DOI:** 10.1107/S160053680904584X

**Published:** 2009-11-07

**Authors:** Long He, Hong-Mei Qin, Lian-Mei Chen

**Affiliations:** a College of Chemistry and Chemical Engineering, China West Normal University, Nanchong 637002, People’s Republic of China; b College of Life Science, China West Normal University, Nanchong 637002, People’s Republic of China

## Abstract

There are two independent mol­ecules in the asymmetric unit of the title compound, C_15_H_12_BrNO_2_. In both mol­ecules, the two benzene rings adopt a *cis* configuration with respect to the ep­oxy ring. In one mol­ecule, the ep­oxy ring makes dihedral angles of 60.5 (2) and 77.92 (19)° with the two benzene rings; in the other mol­ecule, the values are 61.0 (2) and 81.43 (19)°. Inter­molecular N—H⋯O and C—H⋯O hydrogen bonding is present in the crystal structure.

## Related literature

For epoxide-containing compounds used as building blocks in synthesis, see: Diez *et al.* (2008 ▶); Watanabe *et al.* (1998 ▶); Zhu & Espenson (1995 ▶). For related structures, see: He (2009 ▶); He & Chen (2009 ▶).
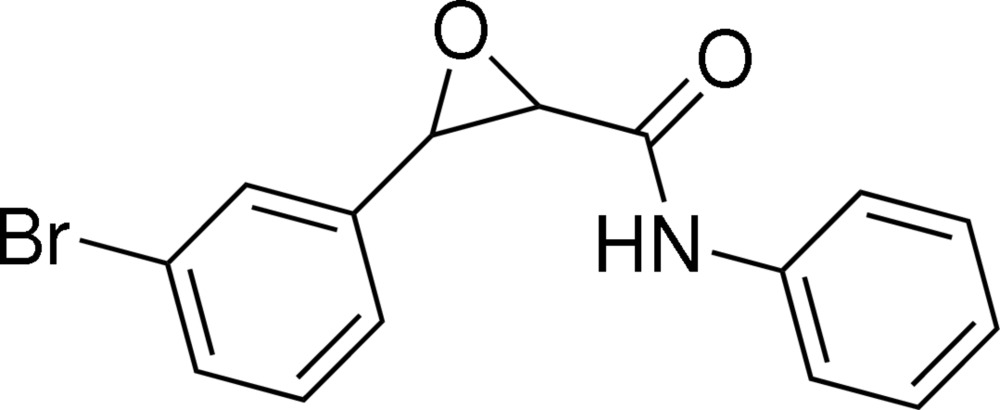


## Experimental

### 

#### Crystal data

C_15_H_12_BrNO_2_
*M* *_r_* = 318.17
Monoclinic, 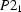

*a* = 5.5124 (1) Å
*b* = 11.1975 (2) Å
*c* = 21.3298 (4) Å
β = 94.405 (2)°
*V* = 1312.69 (4) Å^3^
*Z* = 4
Cu *K*α radiation
μ = 4.25 mm^−1^
*T* = 295 K
0.36 × 0.34 × 0.30 mm

#### Data collection

Oxford Diffraction Gemini S Ultra diffractometer
Absorption correction: multi-scan (*CrysAlis Pro*; Oxford Diffraction, 2009 ▶) *T*_min_ = 0.310, *T*_max_ = 0.362
19177 measured reflections
4142 independent reflections
4027 reflections with *I* > 2σ(*I*)
*R*_int_ = 0.048

#### Refinement

*R*[*F* ^2^ > 2σ(*F* ^2^)] = 0.034
*wR*(*F* ^2^) = 0.084
*S* = 1.00
4142 reflections
351 parameters
17 restraints
H atoms treated by a mixture of independent and constrained refinement
Δρ_max_ = 0.67 e Å^−3^
Δρ_min_ = −0.38 e Å^−3^
Absolute structure: Flack (1983 ▶), 1768 Friedel pairs
Flack parameter: 0.016 (18)

### 

Data collection: *CrysAlis Pro* (Oxford Diffraction, 2009 ▶); cell refinement: *CrysAlis Pro*; data reduction: *CrysAlis Pro*; program(s) used to solve structure: *SHELXS97* (Sheldrick, 2008 ▶); program(s) used to refine structure: *SHELXL97* (Sheldrick, 2008 ▶); molecular graphics: *ORTEP-3* (Farrugia, 1997 ▶); software used to prepare material for publication: *SHELXL97*.

## Supplementary Material

Crystal structure: contains datablocks global, I. DOI: 10.1107/S160053680904584X/xu2663sup1.cif

Structure factors: contains datablocks I. DOI: 10.1107/S160053680904584X/xu2663Isup2.hkl

Additional supplementary materials:  crystallographic information; 3D view; checkCIF report

## Figures and Tables

**Table 1:** Hydrogen-bond geometry (Å, °)

*D*—H⋯*A*	*D*—H	H⋯*A*	*D*⋯*A*	*D*—H⋯*A*
N1—H1*A*⋯O1^i^	0.90 (3)	2.55 (2)	3.359 (4)	150
N2—H2*A*⋯O4^ii^	0.90 (3)	2.53 (2)	3.332 (4)	148
C3—H3⋯O2^iii^	0.93	2.48	3.214 (5)	136
C4—H4⋯O1^i^	0.93	2.36	3.277 (5)	167
C19—H19⋯O4^ii^	0.93	2.27	3.160 (5)	160
C20—H20⋯O3^iv^	0.93	2.51	3.199 (6)	131

Symmetry codes: (i) 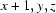; (ii) 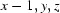; (iii) 
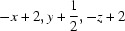
; (iv) 
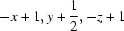
.

**Fig. 1.**
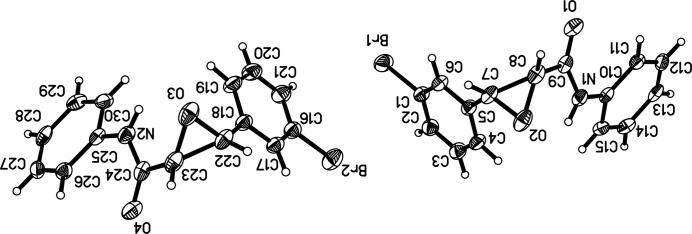
The molecular structure of (I) with 30% probability displacement ellipsoids (arbitrary spheres for H atoms).

## supplementary crystallographic information

### Comment

α, β-Epoxy carbonyl compound are important intermediates for the synthesis of complex molecules. (Diez *et al.* 2008; Watanabe *et al.* 1998). The Darzens reaction, is one of the most powerful method to the synthesis of α, β-epoxy carbonyl and related compounds (Zhu *et al.* 1995). We report herein the crystal structure of the title compound.

The molecular structure of (I) is shown in Fig. 1. Bond lengths and angles in (I) are normal. The asymmetric unit of the title compound consists of two crystallographically independent molecules (Fig. 1) each of which adopts a *cis* configuration about the epoxides ring. The dihedral angle between the C1—C6 and C10—C15 is 86.13 (10)° and that between C16–21 and C25–30 phenyl ring is 83.86 (11)°. O2/C7/C8 epoxide ring makes dihedral angles of 60.47 (22)° and 77.92 (19)° with C6 and C15 phenyl ring, respectively. O3/C22/C23 epoxide ring makes dihedral angles of 60.96 (22)° and 81.43 (19)° with C16 and C25 phenyl ring, respectively. The crystal packing is stabilized by N—H···0 and C—H···0 hydrogen bonding (Table 1).

### Experimental

2-Chloro-*N*-phenylacetamide (0.17 g, 1.0 mmol) and potassium hydroxide (0.112 g, 2.0 mmol) were dissolved in acetonitrile (2 ml). To the solution was added 3-bromophenylaldehyde (0.15 g, 1.0 mmol) at 298 K, the solution was stirred for 60 min and removal of solvent under reduced pressure, the residue was purified through column chromatography. Single crystals suitable for X-ray diffraction were obtained by slow evaporation of an ethyl acetate solution at room temperature for 1 d.

### Refinement

H atoms on N atoms was located in a difference Fourier map and refined isotropically with distance restraints of 0.90±0.01 Å. The carbon-bound hydrogen atoms were placed in calculated positions with C—H = 0.93–0.98 Å and refined using a riding model, *U*_iso_(H) =1.2*U*_eq_(C). The distance restraints of 1.39±0.01 Å were applied for the C—C bonds of the benzene rings.

### Crystal data

**Table:**

C_15_H_12_BrNO_2_	*F*(000) = 640
*M**_r_* = 318.17	*D*_x_ = 1.610 Mg m^−^^3^
Monoclinic, *P*2_1_	Cu *K*α radiation, λ = 1.54184 Å
Hall symbol: P 2yb	Cell parameters from 15364 reflections
*a* = 5.5124 (1) Å	θ = 2.1–71.8°
*b* = 11.1975 (2) Å	µ = 4.25 mm^−^^1^
*c* = 21.3298 (4) Å	*T* = 295 K
β = 94.405 (2)°	Block, colorless
*V* = 1312.69 (4) Å^3^	0.36 × 0.34 × 0.30 mm
*Z* = 4	

### Data collection

**Table:**

Oxford Diffraction Gemini S Ultra diffractometer	4142 independent reflections
Radiation source: Enhance Ultra (Cu) X-ray Source	4027 reflections with *I* > 2σ(*I*)
mirror	*R*_int_ = 0.048
Detector resolution: 15.9149 pixels mm^-1^	θ_max_ = 65.1°, θ_min_ = 2.1°
ω scans	*h* = −6→6
Absorption correction: multi-scan (*CrysAlis PRO*; Oxford Diffraction, 2009)	*k* = −13→10
*T*_min_ = 0.310, *T*_max_ = 0.362	*l* = −25→25
19177 measured reflections	

### Refinement

**Table:**

Refinement on *F*^2^	Secondary atom site location: difference Fourier map
Least-squares matrix: full	Hydrogen site location: inferred from neighbouring sites
*R*[*F*^2^ > 2σ(*F*^2^)] = 0.034	H atoms treated by a mixture of independent and constrained refinement
*wR*(*F*^2^) = 0.084	*w* = 1/[σ^2^(*F*_o_^2^) + (0.0457*P*)^2^ + 0.857*P*] where *P* = (*F*_o_^2^ + 2*F*_c_^2^)/3
*S* = 1.00	(Δ/σ)_max_ = 0.001
4142 reflections	Δρ_max_ = 0.67 e Å^−^^3^
351 parameters	Δρ_min_ = −0.38 e Å^−^^3^
17 restraints	Absolute structure: Flack (1983), 1768 Friedel pairs
Primary atom site location: structure-invariant direct methods	Flack parameter: 0.016 (18)

### Special details

**Table:**

Geometry. All e.s.d.'s (except the e.s.d. in the dihedral angle between two l.s. planes) are estimated using the full covariance matrix. The cell e.s.d.'s are taken into account individually in the estimation of e.s.d.'s in distances, angles and torsion angles; correlations between e.s.d.'s in cell parameters are only used when they are defined by crystal symmetry. An approximate (isotropic) treatment of cell e.s.d.'s is used for estimating e.s.d.'s involving l.s. planes.
Refinement. Refinement of *F*^2^ against ALL reflections. The weighted *R*-factor *wR* and goodness of fit *S* are based on *F*^2^, conventional *R*-factors *R* are based on *F*, with *F* set to zero for negative *F*^2^. The threshold expression of *F*^2^ > σ(*F*^2^) is used only for calculating *R*-factors(gt) *etc*. and is not relevant to the choice of reflections for refinement. *R*-factors based on *F*^2^ are statistically about twice as large as those based on *F*, and *R*- factors based on ALL data will be even larger.

### Fractional atomic coordinates and isotropic or equivalent isotropic displacement parameters (Å^2^)

**Table:**

	*x*	*y*	*z*	*U*_iso_*/*U*_eq_	
Br1	0.42656 (10)	0.89013 (4)	0.78262 (2)	0.07080 (18)	
Br2	0.99441 (11)	0.66221 (5)	0.71660 (2)	0.07210 (17)	
O3	0.7352 (6)	0.2259 (3)	0.52366 (13)	0.0628 (8)	
N2	0.8472 (5)	0.3942 (3)	0.42997 (14)	0.0468 (7)	
N1	0.6413 (5)	0.6562 (3)	1.07453 (13)	0.0453 (7)	
O2	0.7701 (6)	0.4874 (3)	0.98389 (13)	0.0584 (8)	
O1	0.2403 (5)	0.6400 (3)	1.05633 (13)	0.0589 (8)	
O4	1.2515 (5)	0.3825 (3)	0.44653 (13)	0.0592 (7)	
C10	0.6238 (6)	0.7481 (3)	1.12127 (15)	0.0396 (8)	
C17	0.9052 (7)	0.4659 (4)	0.63585 (17)	0.0465 (9)	
H17	1.0499	0.4344	0.6542	0.056*	
C7	0.6277 (7)	0.5369 (4)	0.93584 (17)	0.0457 (9)	
H7	0.5752	0.4803	0.9025	0.055*	
C25	0.8600 (6)	0.4843 (3)	0.38192 (15)	0.0409 (8)	
C13	0.6069 (7)	0.9245 (4)	1.21235 (17)	0.0468 (10)	
H13	0.6094	0.9866	1.2413	0.056*	
C1	0.6161 (7)	0.8207 (4)	0.84397 (17)	0.0466 (9)	
C3	0.9538 (8)	0.8303 (4)	0.90649 (19)	0.0554 (11)	
H3	1.0970	0.8678	0.9216	0.066*	
C5	0.6970 (6)	0.6577 (4)	0.91201 (15)	0.0410 (7)	
C16	0.8251 (7)	0.5765 (4)	0.65623 (18)	0.0489 (9)	
C6	0.5555 (7)	0.7099 (4)	0.86780 (17)	0.0453 (9)	
H6	0.4130	0.6723	0.8522	0.054*	
C8	0.5158 (8)	0.5125 (4)	0.99944 (19)	0.0490 (9)	
H8	0.4108	0.4418	0.9993	0.059*	
C23	0.9853 (8)	0.2580 (4)	0.50714 (18)	0.0509 (9)	
H23	1.0999	0.1910	0.5097	0.061*	
C14	0.8076 (7)	0.9150 (4)	1.16933 (18)	0.0528 (10)	
H14	0.9326	0.9709	1.1736	0.063*	
C4	0.8971 (7)	0.7199 (4)	0.93112 (18)	0.0496 (9)	
H4	1.0035	0.6874	0.9625	0.060*	
C26	1.0499 (7)	0.4918 (4)	0.33747 (17)	0.0460 (9)	
H26	1.1762	0.4365	0.3407	0.055*	
C15	0.8182 (6)	0.8273 (3)	1.12322 (17)	0.0459 (9)	
H15	0.9432	0.8223	1.0964	0.055*	
C11	0.4279 (7)	0.7575 (4)	1.16434 (16)	0.0447 (8)	
H11	0.3017	0.7022	1.1608	0.054*	
C18	0.7812 (6)	0.4049 (4)	0.59137 (16)	0.0445 (9)	
C2	0.8138 (7)	0.8835 (4)	0.86286 (18)	0.0513 (9)	
H2	0.8487	0.9581	0.8465	0.062*	
C21	0.6217 (8)	0.6303 (4)	0.6342 (2)	0.0601 (12)	
H21	0.5726	0.7044	0.6484	0.072*	
C27	1.0480 (7)	0.5783 (4)	0.29070 (17)	0.0475 (10)	
H27	1.1687	0.5828	0.2627	0.057*	
C28	0.8594 (8)	0.6554 (4)	0.28868 (16)	0.0508 (9)	
H28	0.8498	0.7151	0.2583	0.061*	
C9	0.4518 (7)	0.6102 (4)	1.04614 (16)	0.0444 (8)	
C30	0.6630 (6)	0.5628 (4)	0.38105 (18)	0.0490 (9)	
H30	0.5425	0.5588	0.4092	0.059*	
C29	0.6667 (7)	0.6478 (4)	0.33342 (18)	0.0568 (10)	
H29	0.5407	0.7032	0.3294	0.068*	
C20	0.4981 (8)	0.5692 (5)	0.5910 (2)	0.0623 (12)	
H20	0.3527	0.6011	0.5734	0.075*	
C12	0.4244 (7)	0.8461 (4)	1.21031 (16)	0.0505 (11)	
H12	0.3019	0.8503	1.2379	0.061*	
C24	1.0405 (7)	0.3523 (3)	0.45838 (16)	0.0460 (9)	
C19	0.5729 (7)	0.4571 (5)	0.56933 (19)	0.0575 (11)	
H19	0.4740	0.4178	0.5386	0.069*	
C22	0.8683 (8)	0.2850 (4)	0.57056 (18)	0.0499 (9)	
H22	0.9286	0.2333	0.6054	0.060*	
H1A	0.779 (4)	0.631 (3)	1.0586 (16)	0.038 (10)*	
H2A	0.710 (5)	0.369 (4)	0.4461 (18)	0.055 (12)*	

### Atomic displacement parameters (Å^2^)

**Table:**

	*U*^11^	*U*^22^	*U*^33^	*U*^12^	*U*^13^	*U*^23^
Br1	0.0567 (3)	0.0843 (4)	0.0696 (3)	0.0040 (3)	−0.00674 (19)	0.0319 (3)
Br2	0.0783 (3)	0.0641 (3)	0.0704 (3)	0.0098 (3)	−0.0172 (2)	−0.0138 (3)
O3	0.079 (2)	0.0537 (18)	0.0527 (16)	−0.0231 (16)	−0.0157 (14)	0.0082 (14)
N2	0.0472 (16)	0.052 (2)	0.0402 (15)	−0.0079 (18)	−0.0066 (12)	0.0116 (15)
N1	0.0473 (16)	0.0487 (19)	0.0381 (14)	0.0032 (17)	−0.0079 (12)	−0.0073 (15)
O2	0.075 (2)	0.0446 (18)	0.0516 (16)	0.0170 (15)	−0.0173 (14)	−0.0015 (12)
O1	0.0532 (16)	0.063 (2)	0.0580 (16)	0.0031 (15)	−0.0148 (13)	−0.0066 (14)
O4	0.0515 (15)	0.0659 (19)	0.0575 (15)	−0.0072 (17)	−0.0142 (12)	0.0116 (16)
C10	0.0419 (18)	0.040 (2)	0.0352 (17)	0.0040 (15)	−0.0104 (14)	0.0030 (15)
C17	0.0401 (18)	0.051 (2)	0.0463 (19)	0.0012 (17)	−0.0063 (15)	0.0068 (18)
C7	0.052 (2)	0.040 (2)	0.0423 (19)	0.0012 (17)	−0.0148 (16)	−0.0033 (16)
C25	0.0436 (18)	0.040 (2)	0.0369 (17)	−0.0055 (16)	−0.0105 (14)	0.0013 (14)
C13	0.056 (2)	0.044 (2)	0.0374 (18)	0.0146 (18)	−0.0198 (16)	−0.0074 (16)
C1	0.0414 (19)	0.053 (2)	0.0445 (18)	0.0058 (17)	−0.0029 (15)	0.0018 (17)
C3	0.051 (2)	0.064 (3)	0.050 (2)	−0.018 (2)	−0.0049 (18)	−0.0047 (19)
C5	0.0401 (17)	0.045 (2)	0.0374 (16)	0.0005 (18)	−0.0034 (13)	−0.0044 (17)
C16	0.046 (2)	0.052 (2)	0.048 (2)	0.0001 (18)	−0.0019 (16)	0.0050 (17)
C6	0.0415 (19)	0.049 (2)	0.0438 (19)	−0.0016 (16)	−0.0088 (15)	0.0017 (16)
C8	0.061 (2)	0.037 (2)	0.047 (2)	−0.0014 (17)	−0.0124 (17)	0.0010 (16)
C23	0.064 (2)	0.039 (2)	0.047 (2)	−0.0017 (18)	−0.0135 (18)	0.0049 (17)
C14	0.045 (2)	0.050 (3)	0.061 (2)	−0.0024 (17)	−0.0171 (17)	−0.0015 (19)
C4	0.042 (2)	0.061 (3)	0.0439 (19)	−0.0022 (18)	−0.0104 (16)	−0.0056 (18)
C26	0.047 (2)	0.047 (2)	0.0424 (19)	0.0085 (17)	−0.0033 (16)	0.0007 (17)
C15	0.0355 (18)	0.052 (2)	0.049 (2)	0.0028 (16)	−0.0054 (15)	0.0000 (17)
C11	0.0435 (19)	0.047 (2)	0.0422 (18)	−0.0048 (16)	−0.0080 (15)	0.0024 (16)
C18	0.0385 (16)	0.053 (2)	0.0408 (17)	−0.0023 (18)	−0.0057 (13)	0.0110 (17)
C2	0.052 (2)	0.050 (2)	0.0514 (19)	−0.007 (2)	0.0005 (16)	−0.0005 (19)
C21	0.054 (2)	0.063 (3)	0.062 (2)	0.014 (2)	−0.003 (2)	0.011 (2)
C27	0.051 (2)	0.058 (3)	0.0344 (18)	−0.0067 (19)	0.0053 (15)	−0.0034 (17)
C28	0.070 (2)	0.041 (2)	0.0371 (17)	−0.005 (2)	−0.0193 (16)	0.0108 (18)
C9	0.054 (2)	0.041 (2)	0.0368 (17)	0.0025 (16)	−0.0072 (16)	0.0011 (15)
C30	0.0352 (18)	0.058 (3)	0.053 (2)	−0.0027 (17)	−0.0036 (15)	0.0046 (18)
C29	0.0449 (19)	0.058 (3)	0.064 (2)	0.005 (2)	−0.0154 (17)	0.008 (2)
C20	0.046 (2)	0.077 (3)	0.063 (3)	0.013 (2)	−0.0062 (19)	0.015 (2)
C12	0.052 (2)	0.066 (3)	0.0327 (17)	0.017 (2)	−0.0002 (15)	0.0021 (17)
C24	0.058 (2)	0.042 (2)	0.0359 (17)	−0.0058 (17)	−0.0065 (16)	0.0031 (15)
C19	0.043 (2)	0.078 (3)	0.049 (2)	−0.002 (2)	−0.0131 (17)	0.013 (2)
C22	0.058 (2)	0.047 (2)	0.0426 (19)	−0.0070 (18)	−0.0147 (17)	0.0087 (17)

### Geometric parameters (Å, °)

**Table:**

Br1—C1	1.788 (4)	C5—C4	1.341 (5)
Br2—C16	1.807 (4)	C16—C21	1.326 (6)
O3—C22	1.365 (5)	C6—H6	0.9300
O3—C23	1.493 (6)	C8—C9	1.539 (6)
N2—C24	1.274 (5)	C8—H8	0.9800
N2—C25	1.444 (5)	C23—C24	1.528 (5)
N2—H2A	0.90 (3)	C23—C22	1.572 (6)
N1—C9	1.275 (5)	C23—H23	0.9800
N1—C10	1.442 (5)	C14—C15	1.395 (5)
N1—H1A	0.90 (3)	C14—H14	0.9300
O2—C7	1.360 (4)	C4—H4	0.9300
O2—C8	1.492 (5)	C26—C27	1.390 (5)
O1—C9	1.248 (5)	C26—H26	0.9300
O4—C24	1.256 (5)	C15—H15	0.9300
C10—C15	1.389 (5)	C11—C12	1.397 (5)
C10—C11	1.474 (5)	C11—H11	0.9300
C17—C18	1.317 (5)	C18—C19	1.341 (5)
C17—C16	1.396 (6)	C18—C22	1.504 (6)
C17—H17	0.9300	C2—H2	0.9300
C7—C5	1.505 (6)	C21—C20	1.298 (7)
C7—C8	1.557 (6)	C21—H21	0.9300
C7—H7	0.9800	C27—C28	1.349 (5)
C25—C30	1.396 (5)	C27—H27	0.9300
C25—C26	1.468 (5)	C28—C29	1.484 (5)
C13—C12	1.333 (6)	C28—H28	0.9300
C13—C14	1.494 (5)	C30—C29	1.393 (5)
C13—H13	0.9300	C30—H30	0.9300
C1—C2	1.333 (5)	C29—H29	0.9300
C1—C6	1.392 (6)	C20—C19	1.411 (7)
C3—C2	1.306 (6)	C20—H20	0.9300
C3—C4	1.388 (6)	C12—H12	0.9300
C3—H3	0.9300	C19—H19	0.9300
C5—C6	1.314 (5)	C22—H22	0.9800
			
C22—O3—C23	66.6 (3)	C13—C14—H14	118.1
C24—N2—C25	120.7 (3)	C5—C4—C3	123.5 (4)
C24—N2—H2A	113 (3)	C5—C4—H4	118.3
C25—N2—H2A	125 (3)	C3—C4—H4	118.3
C9—N1—C10	121.4 (3)	C27—C26—C25	122.5 (3)
C9—N1—H1A	112 (2)	C27—C26—H26	118.8
C10—N1—H1A	126 (3)	C25—C26—H26	118.8
C7—O2—C8	66.0 (3)	C10—C15—C14	113.3 (3)
C15—C10—N1	112.6 (3)	C10—C15—H15	123.3
C15—C10—C11	122.5 (3)	C14—C15—H15	123.3
N1—C10—C11	124.9 (3)	C12—C11—C10	122.6 (3)
C18—C17—C16	121.7 (4)	C12—C11—H11	118.7
C18—C17—H17	119.2	C10—C11—H11	118.7
C16—C17—H17	119.2	C17—C18—C19	114.2 (4)
O2—C7—C5	118.3 (3)	C17—C18—C22	121.0 (3)
O2—C7—C8	61.1 (3)	C19—C18—C22	124.7 (4)
C5—C7—C8	125.5 (3)	C3—C2—C1	113.5 (4)
O2—C7—H7	113.9	C3—C2—H2	123.2
C5—C7—H7	113.9	C1—C2—H2	123.2
C8—C7—H7	113.9	C20—C21—C16	113.3 (5)
C30—C25—N2	111.6 (3)	C20—C21—H21	123.3
C30—C25—C26	123.2 (3)	C16—C21—H21	123.3
N2—C25—C26	125.1 (3)	C28—C27—C26	115.6 (4)
C12—C13—C14	121.7 (4)	C28—C27—H27	122.2
C12—C13—H13	119.1	C26—C27—H27	122.2
C14—C13—H13	119.1	C27—C28—C29	122.1 (4)
C2—C1—C6	125.0 (4)	C27—C28—H28	119.0
C2—C1—Br1	114.2 (3)	C29—C28—H28	119.0
C6—C1—Br1	120.8 (3)	O1—C9—N1	123.5 (4)
C2—C3—C4	122.6 (4)	O1—C9—C8	124.5 (4)
C2—C3—H3	118.7	N1—C9—C8	112.0 (4)
C4—C3—H3	118.7	C29—C30—C25	112.6 (3)
C6—C5—C4	114.6 (4)	C29—C30—H30	123.7
C6—C5—C7	119.4 (3)	C25—C30—H30	123.7
C4—C5—C7	126.0 (3)	C30—C29—C28	124.0 (4)
C21—C16—C17	124.9 (4)	C30—C29—H29	118.0
C21—C16—Br2	112.8 (3)	C28—C29—H29	118.0
C17—C16—Br2	122.3 (3)	C21—C20—C19	123.4 (4)
C5—C6—C1	120.9 (4)	C21—C20—H20	118.3
C5—C6—H6	119.6	C19—C20—H20	118.3
C1—C6—H6	119.6	C13—C12—C11	116.1 (4)
O2—C8—C9	123.0 (3)	C13—C12—H12	122.0
O2—C8—C7	53.0 (2)	C11—C12—H12	122.0
C9—C8—C7	124.4 (3)	O4—C24—N2	123.9 (4)
O2—C8—H8	114.3	O4—C24—C23	124.0 (4)
C9—C8—H8	114.3	N2—C24—C23	112.1 (4)
C7—C8—H8	114.3	C18—C19—C20	122.5 (4)
O3—C23—C24	124.2 (3)	C18—C19—H19	118.8
O3—C23—C22	52.8 (2)	C20—C19—H19	118.8
C24—C23—C22	124.7 (4)	O3—C22—C18	118.9 (3)
O3—C23—H23	113.9	O3—C22—C23	60.6 (3)
C24—C23—H23	113.9	C18—C22—C23	125.9 (3)
C22—C23—H23	113.9	O3—C22—H22	113.6
C15—C14—C13	123.8 (4)	C18—C22—H22	113.6
C15—C14—H14	118.1	C23—C22—H22	113.6
			
C9—N1—C10—C15	−148.8 (4)	Br1—C1—C2—C3	−178.3 (3)
C9—N1—C10—C11	33.1 (5)	C17—C16—C21—C20	0.5 (7)
C8—O2—C7—C5	117.1 (4)	Br2—C16—C21—C20	−179.7 (4)
C24—N2—C25—C30	−147.1 (4)	C25—C26—C27—C28	−0.1 (6)
C24—N2—C25—C26	34.9 (5)	C26—C27—C28—C29	−0.1 (6)
O2—C7—C5—C6	−176.6 (4)	C10—N1—C9—O1	−0.2 (6)
C8—C7—C5—C6	−103.5 (4)	C10—N1—C9—C8	−178.9 (3)
O2—C7—C5—C4	4.3 (6)	O2—C8—C9—O1	173.9 (4)
C8—C7—C5—C4	77.4 (5)	C7—C8—C9—O1	109.1 (5)
C18—C17—C16—C21	0.5 (7)	O2—C8—C9—N1	−7.5 (5)
C18—C17—C16—Br2	−179.3 (3)	C7—C8—C9—N1	−72.2 (5)
C4—C5—C6—C1	0.2 (6)	N2—C25—C30—C29	−177.7 (3)
C7—C5—C6—C1	−179.0 (3)	C26—C25—C30—C29	0.3 (5)
C2—C1—C6—C5	−0.6 (6)	C25—C30—C29—C28	−0.5 (6)
Br1—C1—C6—C5	178.7 (3)	C27—C28—C29—C30	0.5 (7)
C7—O2—C8—C9	−110.7 (4)	C16—C21—C20—C19	−0.3 (7)
C5—C7—C8—O2	−105.8 (4)	C14—C13—C12—C11	−2.0 (6)
O2—C7—C8—C9	108.0 (4)	C10—C11—C12—C13	1.4 (6)
C5—C7—C8—C9	2.3 (6)	C25—N2—C24—O4	−1.6 (6)
C22—O3—C23—C24	−110.4 (4)	C25—N2—C24—C23	−179.8 (3)
C12—C13—C14—C15	1.7 (6)	O3—C23—C24—O4	176.9 (4)
C6—C5—C4—C3	−0.3 (6)	C22—C23—C24—O4	111.6 (5)
C7—C5—C4—C3	178.8 (4)	O3—C23—C24—N2	−4.9 (6)
C2—C3—C4—C5	0.9 (7)	C22—C23—C24—N2	−70.1 (5)
C30—C25—C26—C27	0.0 (6)	C17—C18—C19—C20	1.7 (6)
N2—C25—C26—C27	177.7 (4)	C22—C18—C19—C20	179.2 (4)
N1—C10—C15—C14	−178.4 (3)	C21—C20—C19—C18	−0.8 (7)
C11—C10—C15—C14	−0.2 (5)	C23—O3—C22—C18	117.3 (4)
C13—C14—C15—C10	−0.4 (5)	C17—C18—C22—O3	−179.1 (4)
C15—C10—C11—C12	−0.3 (5)	C19—C18—C22—O3	3.5 (6)
N1—C10—C11—C12	177.7 (3)	C17—C18—C22—C23	−106.1 (4)
C16—C17—C18—C19	−1.5 (6)	C19—C18—C22—C23	76.5 (6)
C16—C17—C18—C22	−179.1 (4)	C24—C23—C22—O3	109.5 (4)
C4—C3—C2—C1	−1.1 (6)	O3—C23—C22—C18	−106.1 (4)
C6—C1—C2—C3	1.0 (6)	C24—C23—C22—C18	3.4 (6)

### Hydrogen-bond geometry (Å, °)

**Table:**

*D*—H···*A*	*D*—H	H···*A*	*D*···*A*	*D*—H···*A*
N1—H1A···O1^i^	0.90 (3)	2.55 (2)	3.359 (4)	150
N2—H2A···O4^ii^	0.90 (3)	2.53 (2)	3.332 (4)	148
C3—H3···O2^iii^	0.93	2.48	3.214 (5)	136
C4—H4···O1^i^	0.93	2.36	3.277 (5)	167
C19—H19···O4^ii^	0.93	2.27	3.160 (5)	160
C20—H20···O3^iv^	0.93	2.51	3.199 (6)	131

Symmetry codes: (i) *x*+1, *y*, *z*; (ii) *x*−1, *y*, *z*; (iii) −*x*+2, *y*+1/2, −*z*+2; (iv) −*x*+1, *y*+1/2, −*z*+1.
